# Use of Extract of Bullock’s Blood

**Published:** 1854-05

**Authors:** 


Use of Extract of Bullock's Blood.—Dr. Behrend thus writes: "Dr. Mauthner, to whom we are indebted for the introduction of this remedy, answers a request of mine as follows:—'I now give it to children in larger doses than before, to the extent of half an ounce in a day, dissolved in water. In many anæmic states the favorable result is so striking that the parents, perceiving the improvement of their child, generally desire a continuance of the agent. In these larger doses, it is true, the drug colors the dejections of a brown hue, but it does not give rise to the least dyspeptic symptom. It has never caused emesis, and if the child has shown some dislike to it at first, it takes it afterwards with great avidity. Children who were in the extreme stage of exhaustion, whose stomachs were so irritable that milk and beef-tea or broth were rejected by them, and cod-liver oil could not be in the least retained, bore the extract of ox-blood well, and throve admirably.' Here, in Berlin, [Mauthner is at Vienna,] the extractum bovini is given with very good effect to chlorotic and emaciated girls, and even to phthisical adults. A colleague has found it very efficacious in rachitis."—*Roger, in Jour. f. Kinderkhtn.*

